# Supplementary material for: Urban community health workers in Punjab, India: A qualitative study of ASHAs’ roles in the health system
Source: PLOS Glob Public Health. 2025 Aug 14;5(8):e0004629. doi: 10.1371/journal.pgph.0004629 (PMC12352748; doi:10.1371/journal.pgph.0004629)
Supplement: Attachment — Submitted filename: Response to Reviewers_17July2025.docx [file pgph.0004629.s002.docx]

**1. Please upload a copy of Table 1 which you refer to in your text on page 21.**

This has been uploaded in the manuscript of the text.
